# Supplementary figures and images for: Inactivation of Invs/Nphp2 in renal epithelial cells drives infantile nephronophthisis like phenotypes in mouse
Source: eLife. 2023 Mar 15;12:e82395. doi: 10.7554/eLife.82395 (PMC10154023; doi:10.7554/eLife.82395)

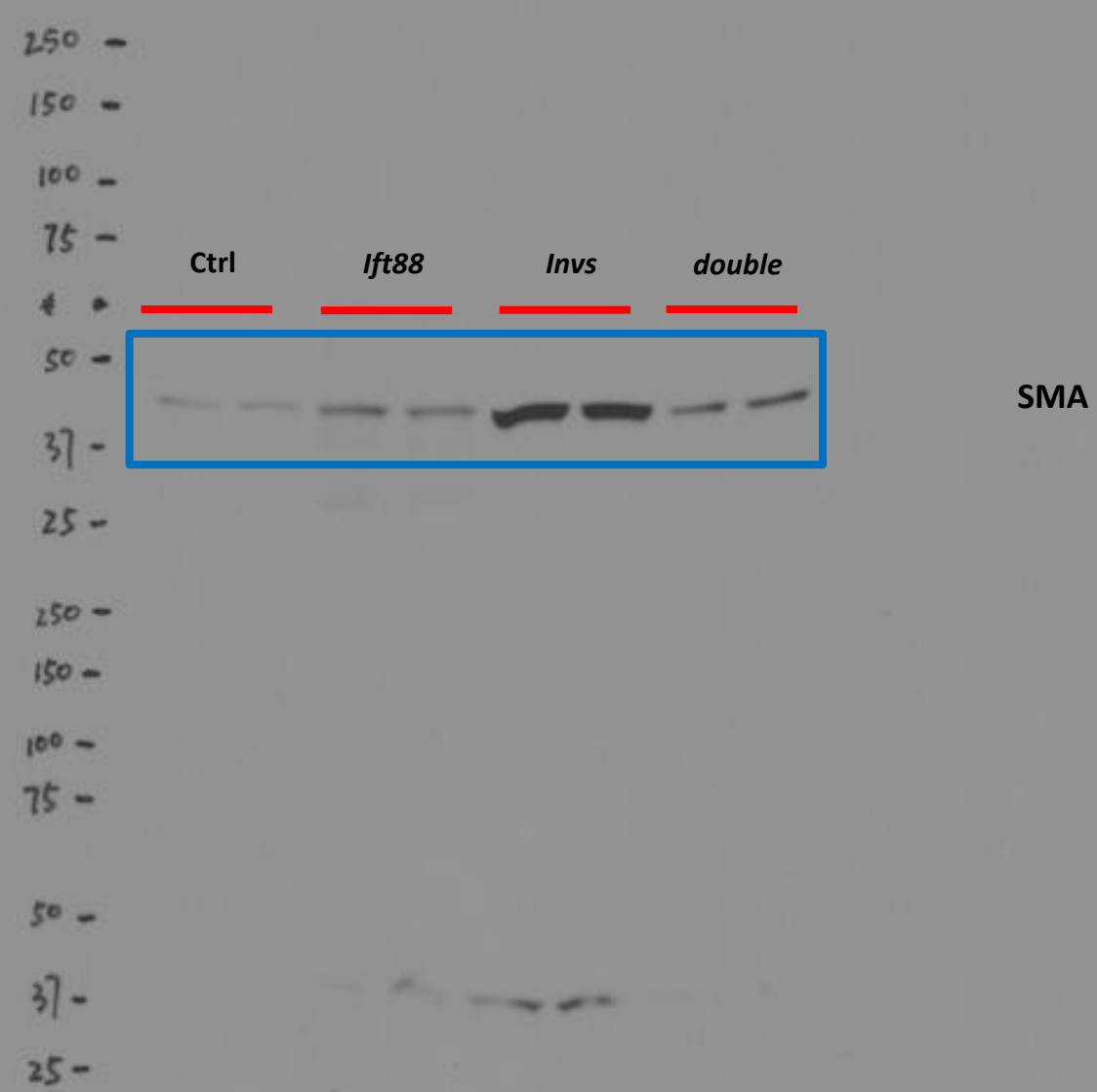

7.5% gel SMA

2

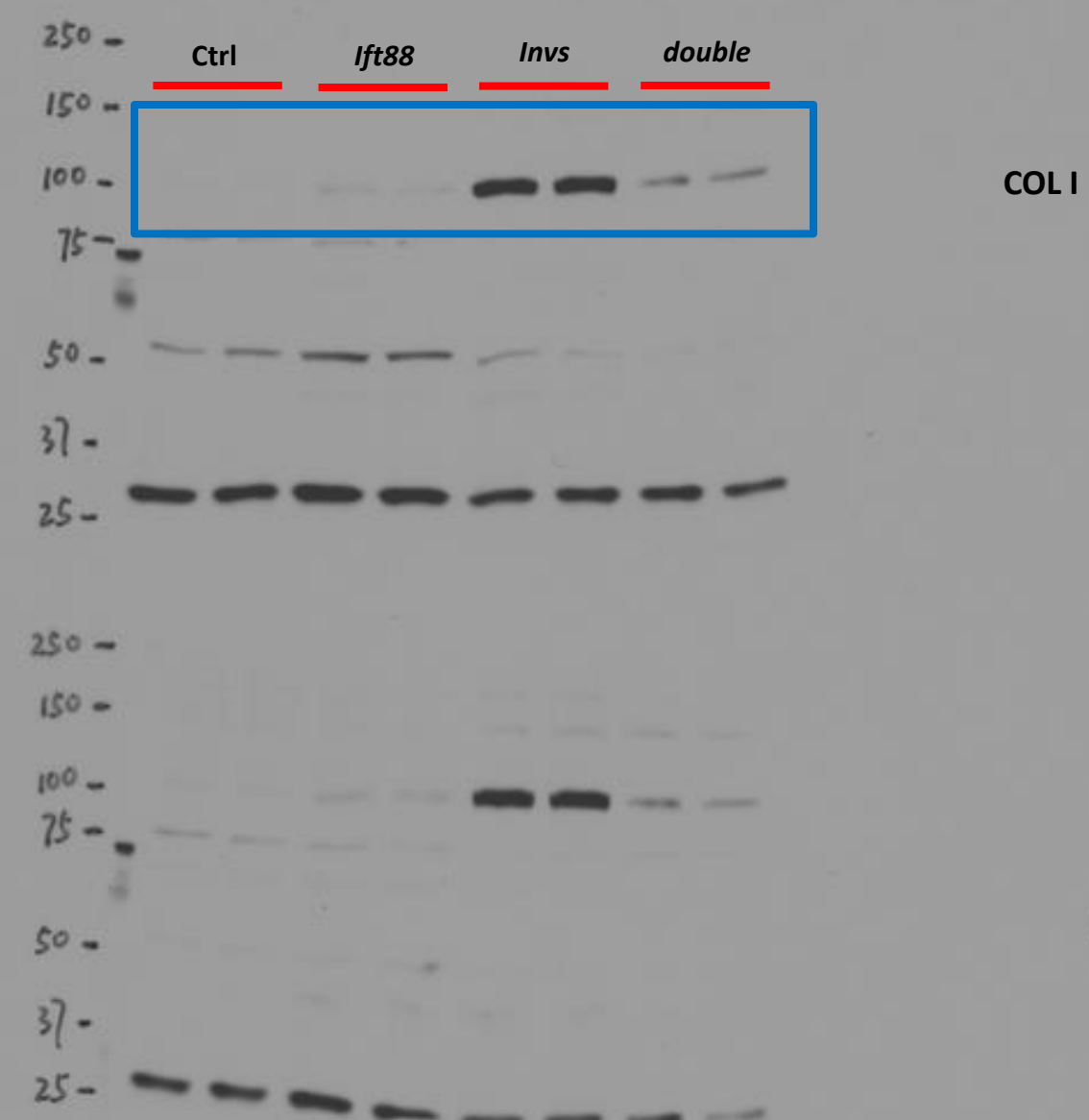

7.5% gel COL1

4

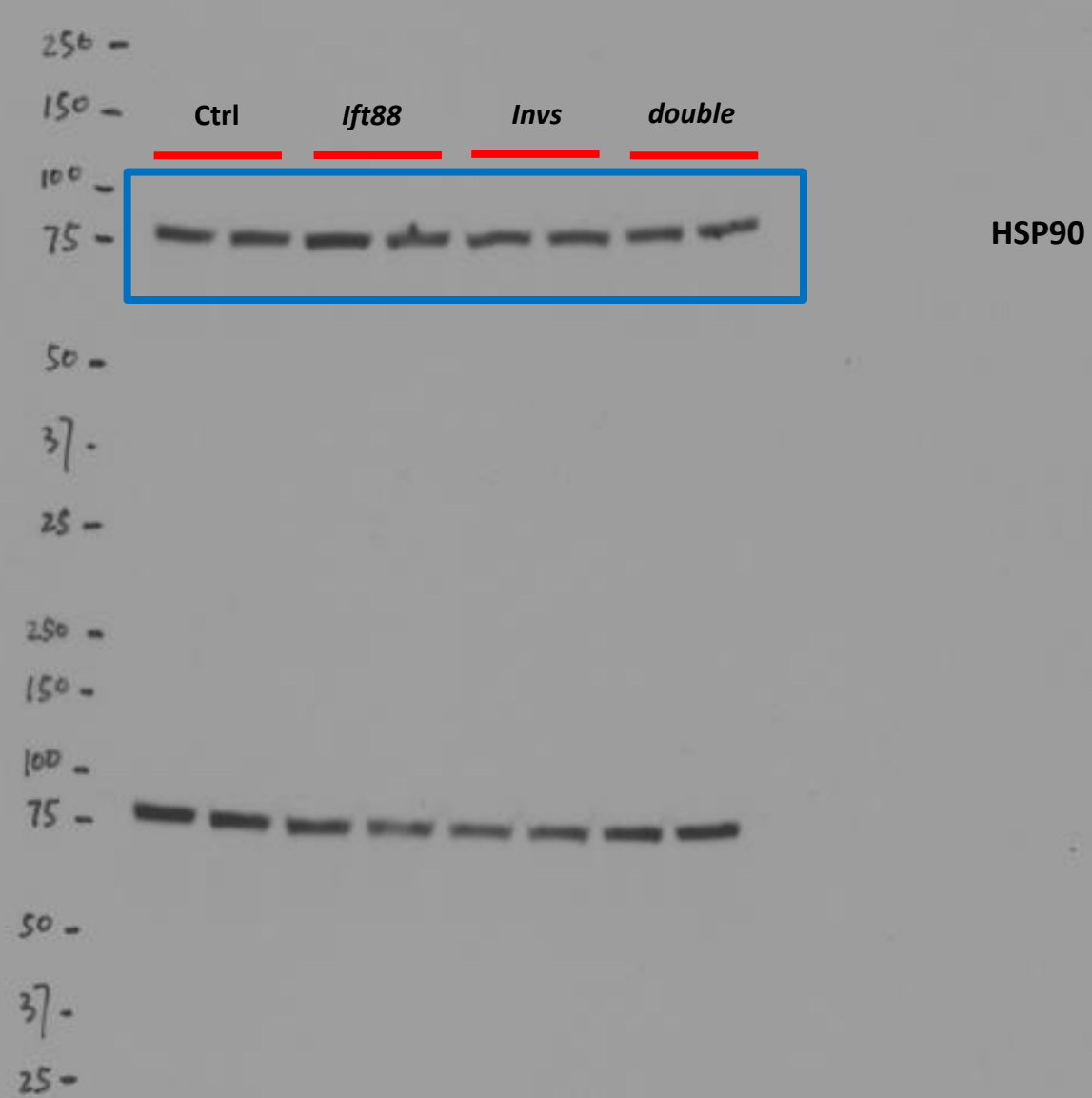

7.5% HSP90

2

Supplement: Figure 5—source data 1. — Littermates are highlighted by the same color in data tables. [file elife-82395-fig5-data1.zip › Fig 5 Source data/Fig 5J Western Blot/Fig 5J.pdf]

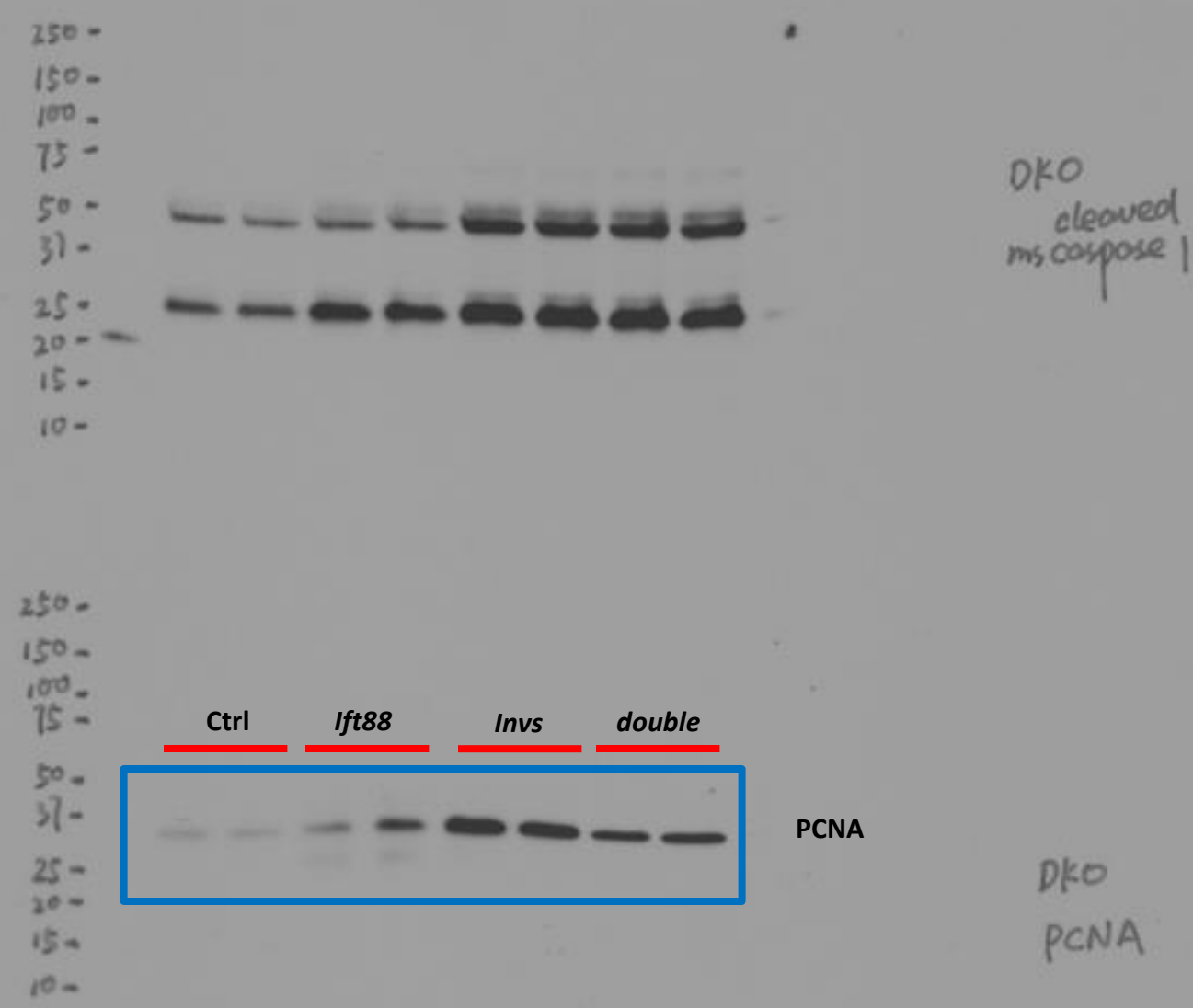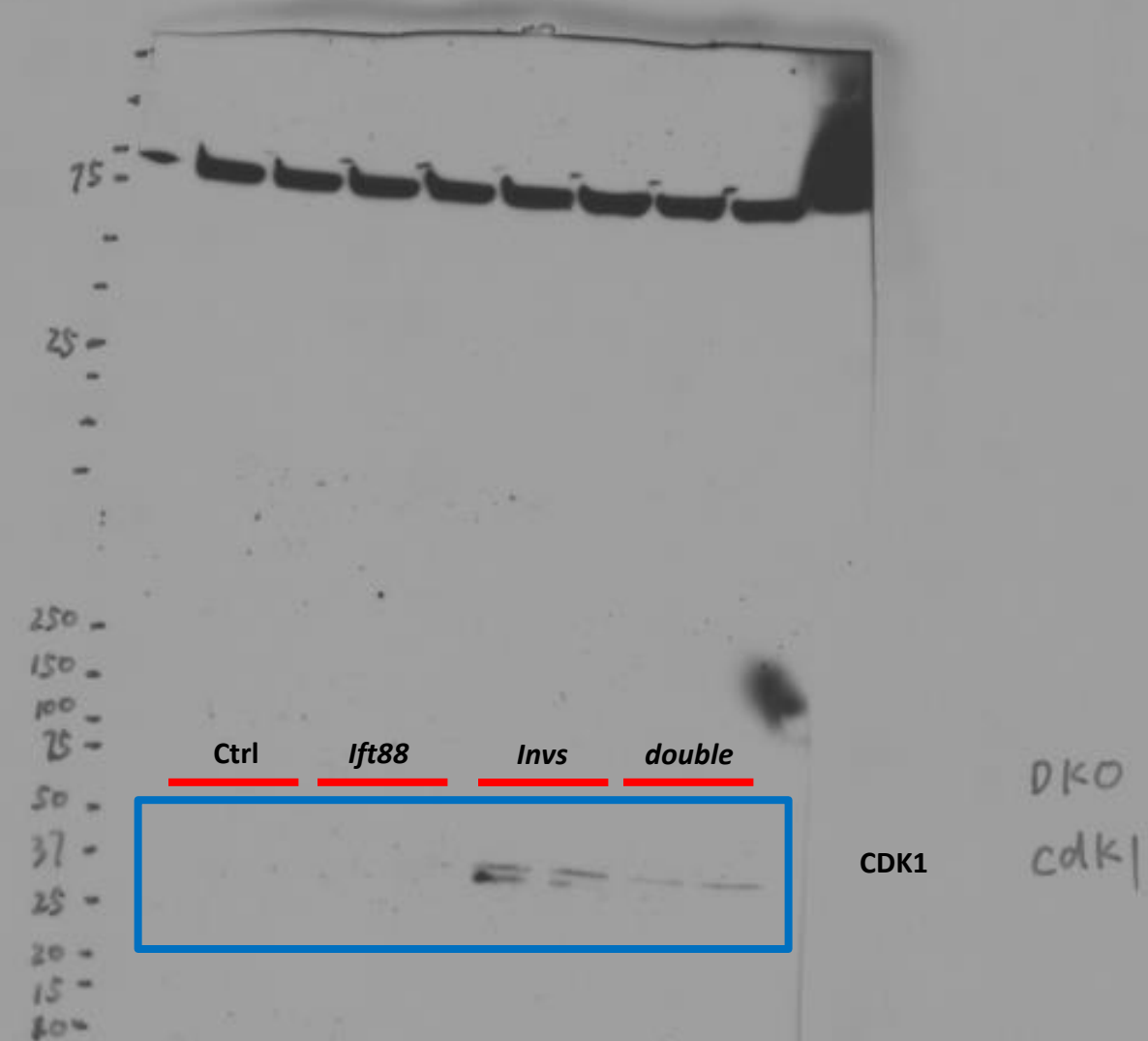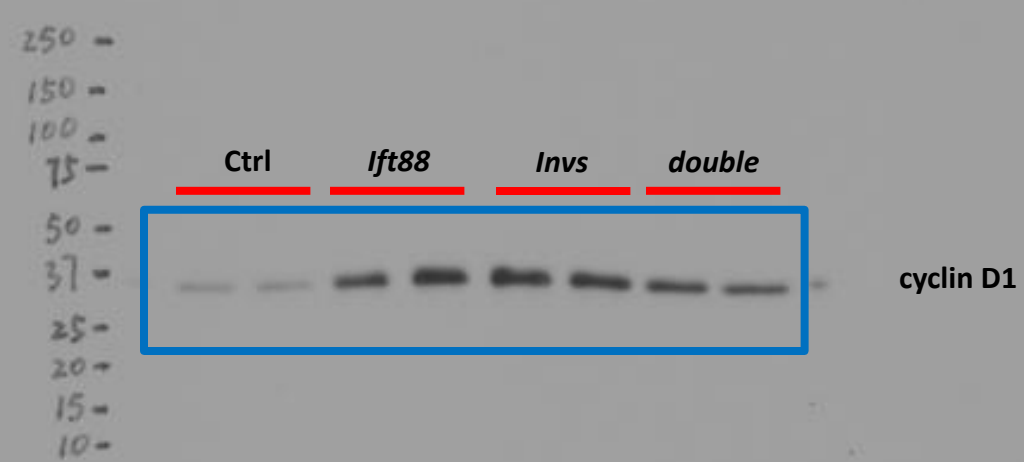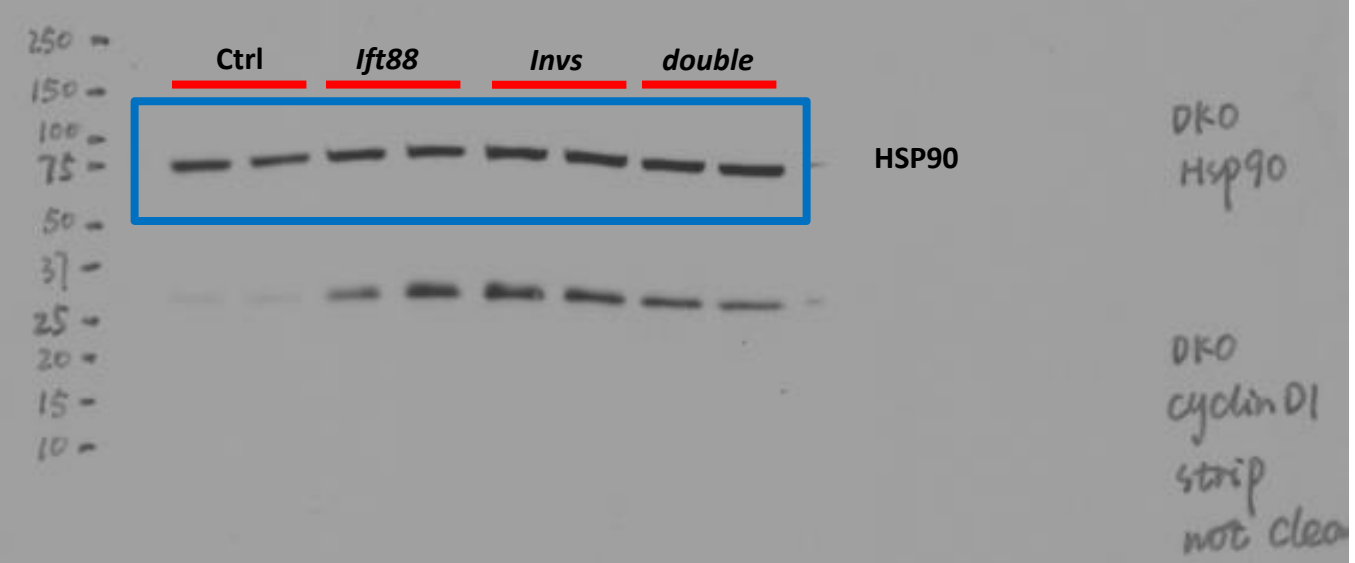

Supplement: Figure 6—source data 1. [file elife-82395-fig6-data1.zip › Fig 6 Source data/Fig 6C Western blot/Fig 6C.pdf]

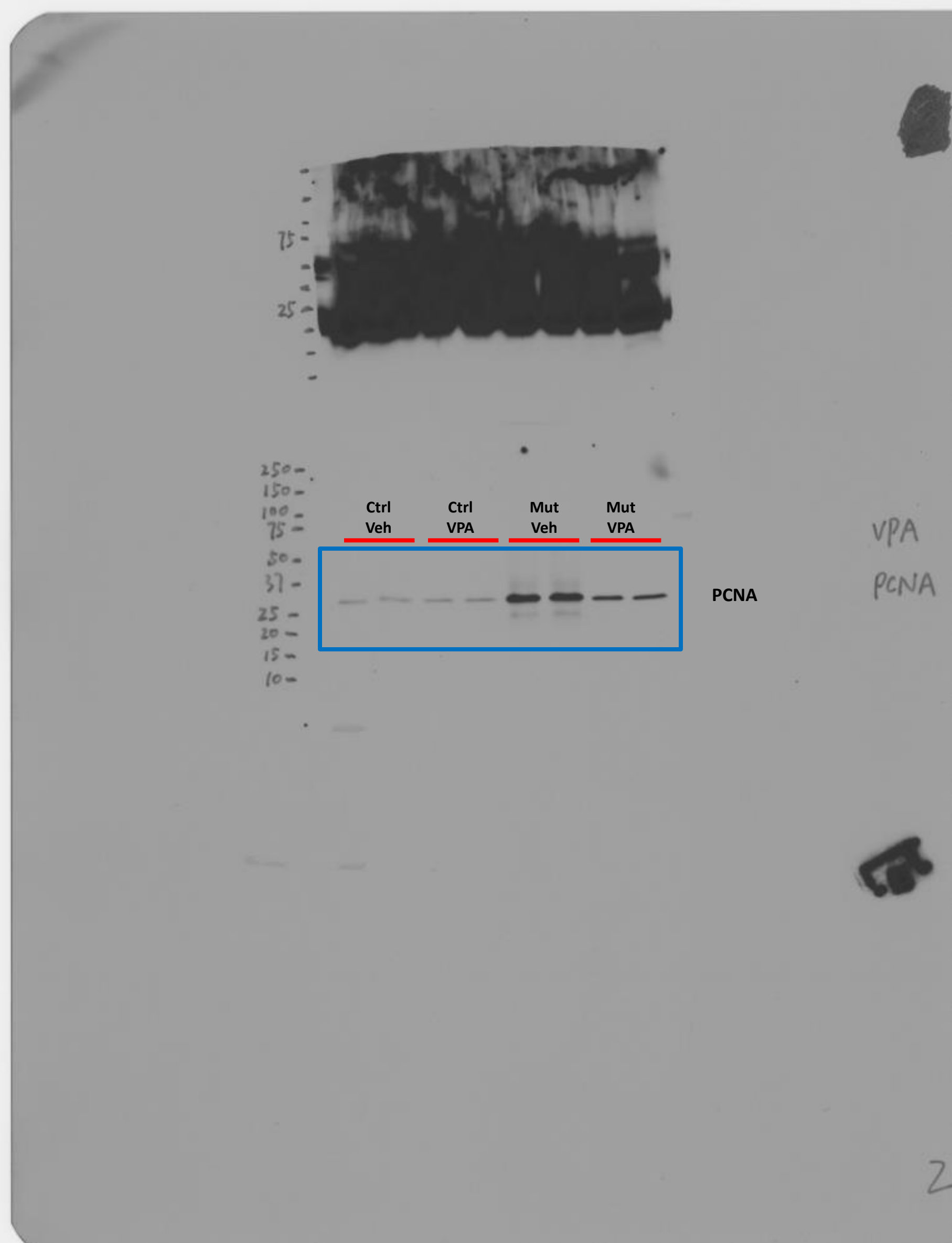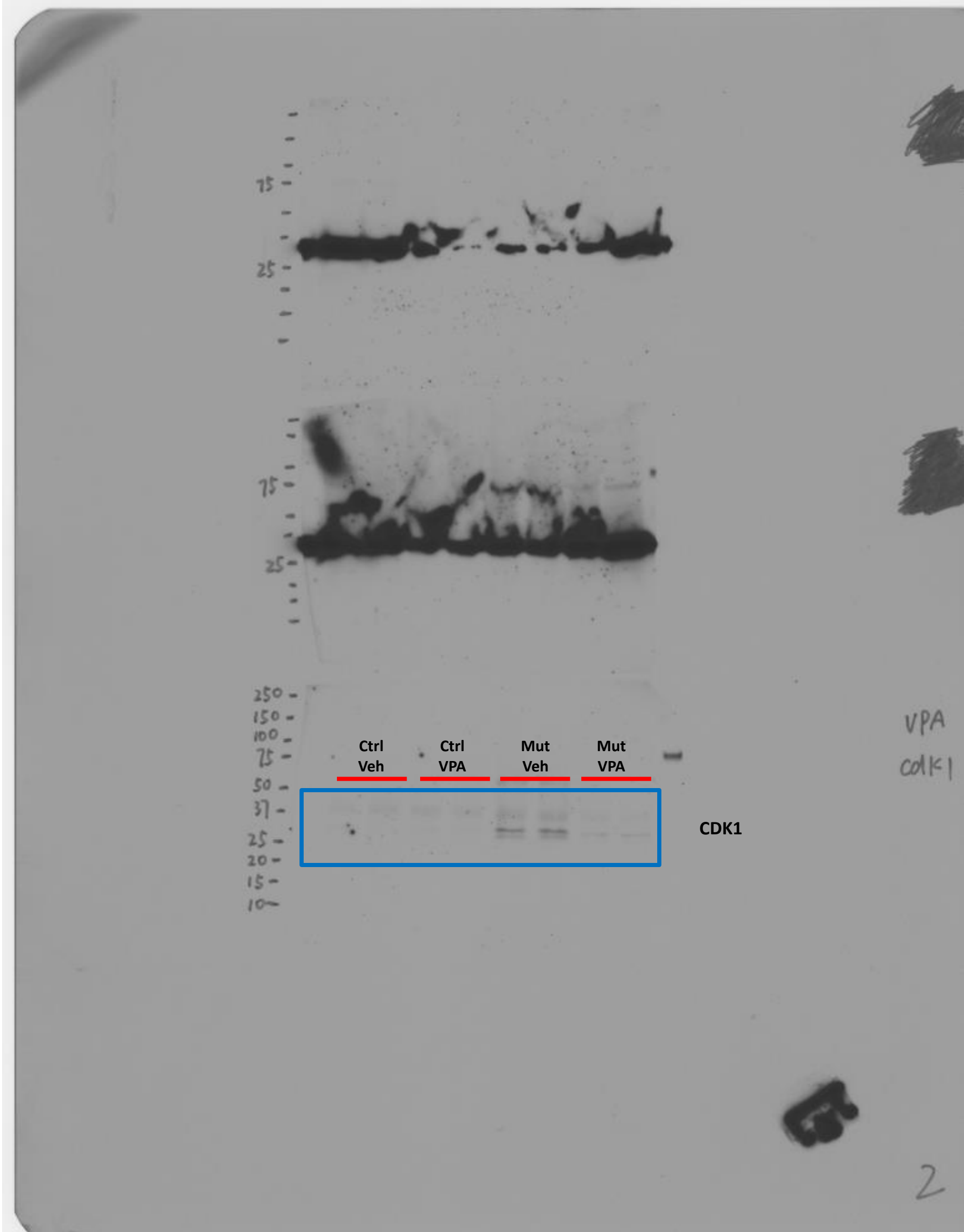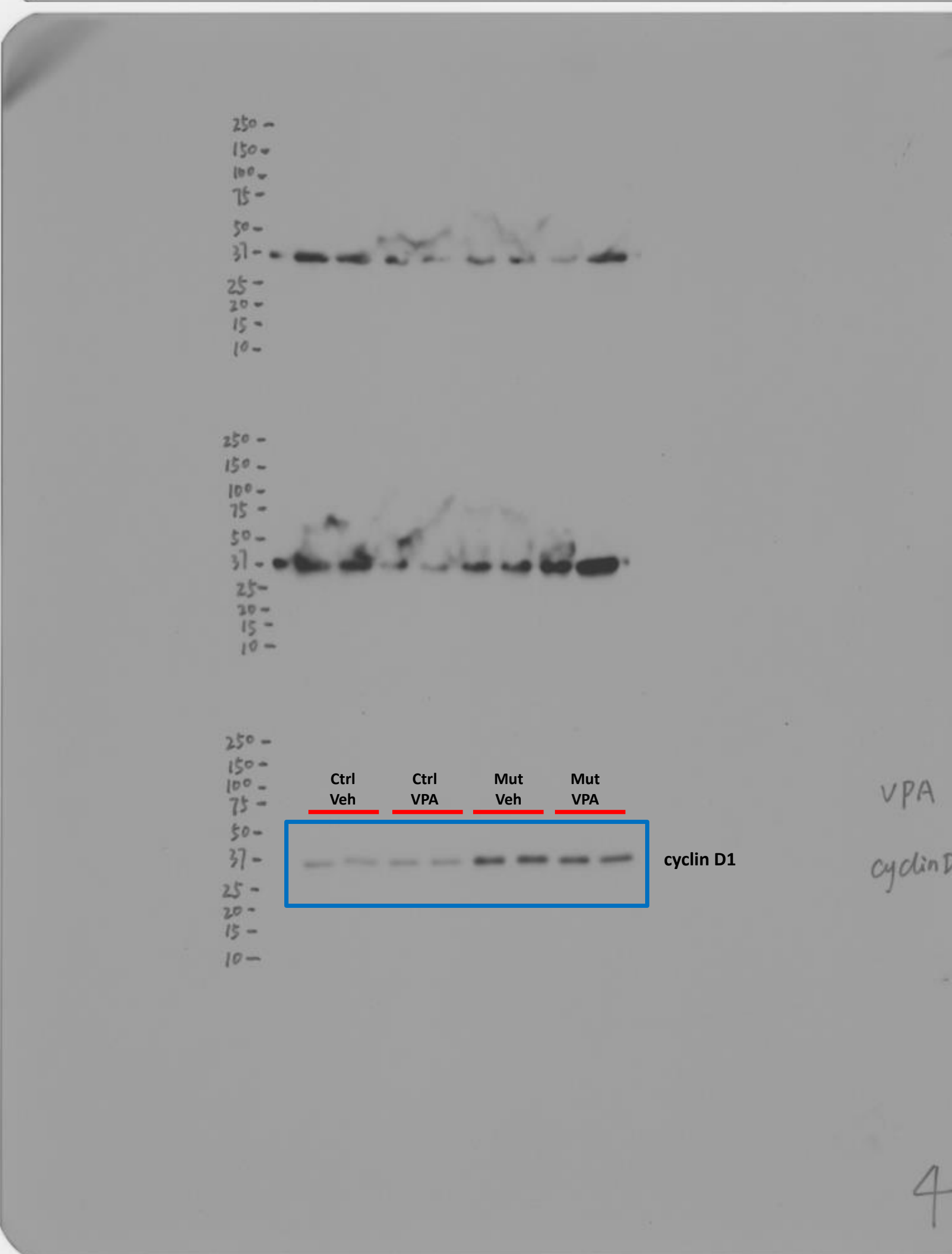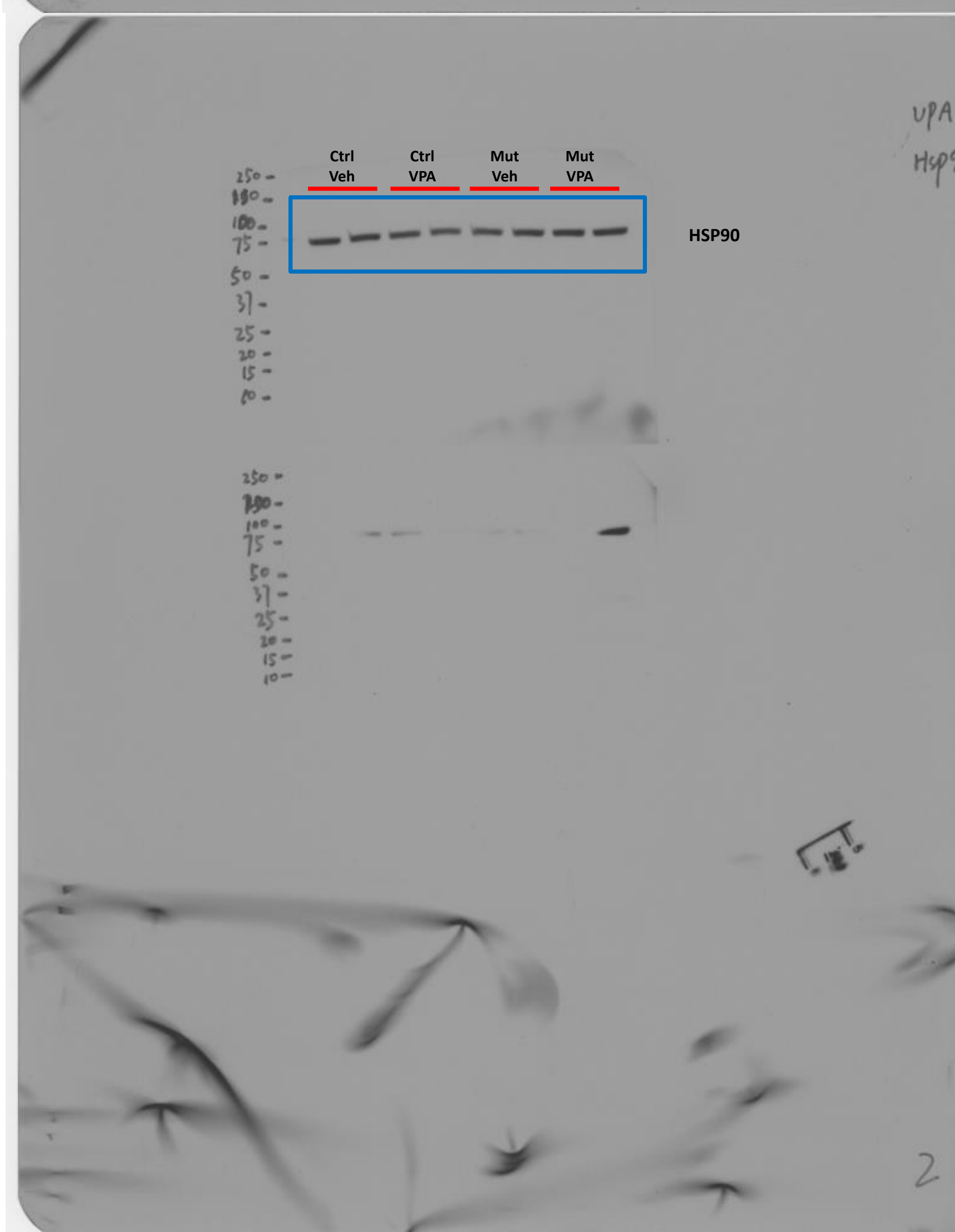

Supplement: Figure 7—source data 1. — Littermates are highlighted by the same color in data tables. [file elife-82395-fig7-data1.zip › Fig 7 Source data/Fig 7I Western blot/Fig 7I.pdf]
