# Supplementary material for: Inactivation of Invs/Nphp2 in renal epithelial cells drives infantile nephronophthisis like phenotypes in mouse
Source: eLife. 2023 Mar 15;12:e82395. doi: 10.7554/eLife.82395 (PMC10154023; doi:10.7554/eLife.82395)
Supplement: Supplementary file 1. [file elife-82395-supp1.docx]

**Supplementary File 1: Sequences of oligos used in this study.**

| Oligo name | Oligo sequence 5’-3’ |
| --- | --- |
| *Invs flox_F* | AGGTTAGCAGCTGGGCAGGAT |
| *Invs flox_R* | TGAGGTAGA CAGTAGCATTCCTGC |
| *Ift88 flox_F* | GACCACCTTTTTAGCCTCCTG |
| *Ift88 flox_R* | AGGGAAGGGACTTAGGAATGA |
| *Cdh16-Cre* carrier_F | CAAATGTTGCTTGTCTGGTG |
| *Cdh16-Cre* carrier_R | GTCAGTCGAGTGCACAGTTT |
| *Cdh16-Cre*  non-carrier_F | AGGCAAATTTTGGTGTACGG |
| *Cdh16-Cre*  non-carrier_R | GCAGATCTGGCTCTCCAAAG |
| *Pkhd1-Cre*_F | CTGGTTGTCATTGGCCAGG |
| *Pkhd1-Cre*_R | GCATCGACCGGTAATGCAGGC |
| *Foxd1-Cre* Common | TCTGGTCCAAGAATCCGAAG |
| *Foxd1-Cre* wildtype Forward | CTCCTCCGTGTCCTCGTC |
| *Foxd1-Cre*  mutant Forward | GGGAGGATTGGGAAGACAAT |
| *Sma* QPCR_F | CGTCCCAGACATCAGGGAGTA |
| *Sma* QPCR_R | ATAGCCACATACATGGCGGG |
| *Vimentin* QPCR_F | ACCCTGCAGTCATTCAGACA |
| *Vimentin* QPCR_R | CAGAGAGGTCAGCAAACTTGGAC |
